# Respect for Autonomy in Patients with Altered Reality Judgment

**DOI:** 10.1192/j.eurpsy.2025.1511

**Published:** 2025-08-26

**Authors:** M. F. Tascon Guerra, E. Ramos Garcia, M. V. Lopez Rodrigo, Y. D’hiver Cantalejo, M. Palomo Monje

**Affiliations:** 1 Psychiatry, Hospital Ntra Sra del Prado, Talavera de la Reina; 2 Psychiatry, SESCAM, Talavera de la Rein, Spain

## Abstract

**Introduction:**

Bioethics is a discipline based on ethical principles aimed at guiding healthcare practice. Four fundamental principles are defined: autonomy, non-maleficence, beneficence, and justice. The first of these is autonomy, which expresses the ability of each individual to make decisions regarding their own health.

**Objectives:**

This case presents a 47-year-old male patient diagnosed with schizophrenia, legally incapacitated and under guardianship by a Foundation. He has no awareness of his illness, is on pharmacological treatment in a controlled environment, and does not cooperate for testing or procedures. He has been involuntarily admitted to the Psychiatry Unit since 2022, awaiting transfer to a residential facility.

**Methods:**

In recent months, the patient has exhibited dysphagia and constitutional syndrome. A tumor suspicious for malignant oropharyngeal neoplasm is identified. He is informed in simple terms, in the presence of his legal guardians, that he has a tumor with malignant characteristics, which will grow over time, eventually blocking his airway and leading to death. Testing is necessary to reach a diagnosis and propose treatment.

**Results:**

The patient repeatedly and firmly expresses his desire not to undergo any tests or treatment. In coordination with the Psychiatry and Otorhinolaryngology services, and in consultation with the Bioethics Committee, it is decided to respect the patient’s autonomy and his decision to refuse tests or invasive procedures. At all times, comfort measures and pain management are provided.

**Conclusions:**

Autonomy is the ability to have control over one’s own life. Every person has the right to make decisions about their health. Mental impairments can alter a person’s autonomy, as they hinder the conscious process of decision-making.

The dignity of the person plays a key role in the protection of life, physical and psychological integrity, and the freedom of individuals with mental disorders.

**Disclosure of Interest:**

None Declared

